# Supplementary material for: Diagnosis and treatment of digestive cancers during COVID-19 in Japan: A Cancer Registry-based Study on the Impact of COVID-19 on Cancer Care in Osaka (CanReCO)
Source: PLoS One. 2022 Sep 20;17(9):e0274918. doi: 10.1371/journal.pone.0274918 (PMC9488819; doi:10.1371/journal.pone.0274918)
Supplement: S1 Fig — (PDF) [file pone.0274918.s001.pdf]

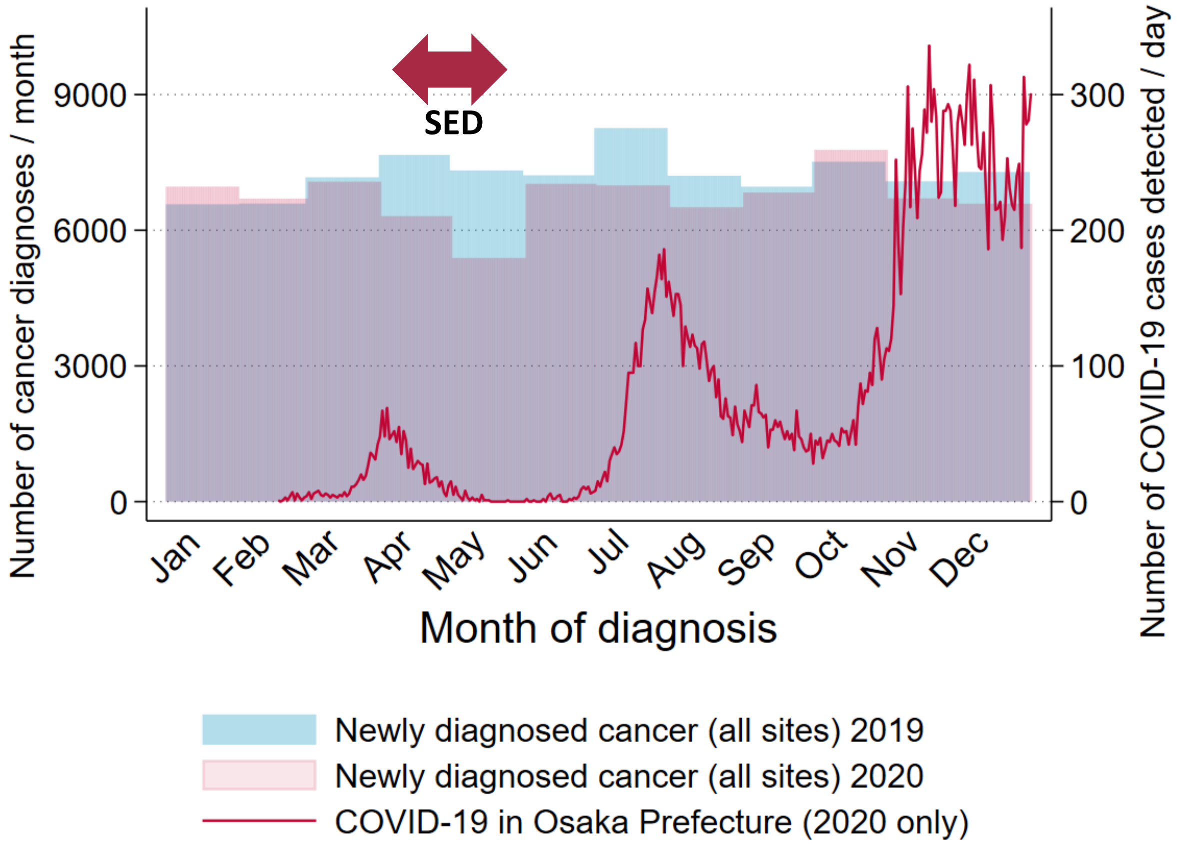

S1 Fig. Number of diagnoses (all sites) in the CanReCO project and number of COVID-19 cases detected, Osaka, Japan, 2019 and 2020.

Abbreviations: SED, state of emergency declaration. The total number of diagnoses in the CanReCO project (all sites combined) was 86 857 in 2019 and 80 869 in 2020 (excluding records with second opinions, relative change -6.9%). The SED was enforced on 7 April 2020 and lifted on 21 May 2020 in Osaka Prefecture [1].
Source for COVID-19: Osaka Prefectural Government. [Surveillance of COVID-19 infection, Osaka Prefecture (in Japanese)]. Osaka: 2021 [cited 2021 Nov 14]. Available from: https://covid19-osaka.info/.

1. Cabinet Secretariat. [State of emergency declaration (issued on 2020 April 7) (in Japanese)]. Tokyo: 2020 [cited 2022 May 11]. Available from: https://corona.go.jp/emergency/.
