# Supplementary material for: Diagnosis and treatment of digestive cancers during COVID-19 in Japan: A Cancer Registry-based Study on the Impact of COVID-19 on Cancer Care in Osaka (CanReCO)
Source: PLoS One. 2022 Sep 20;17(9):e0274918. doi: 10.1371/journal.pone.0274918 (PMC9488819; doi:10.1371/journal.pone.0274918)
Supplement: S2 Fig — (PDF) [file pone.0274918.s002.pdf]

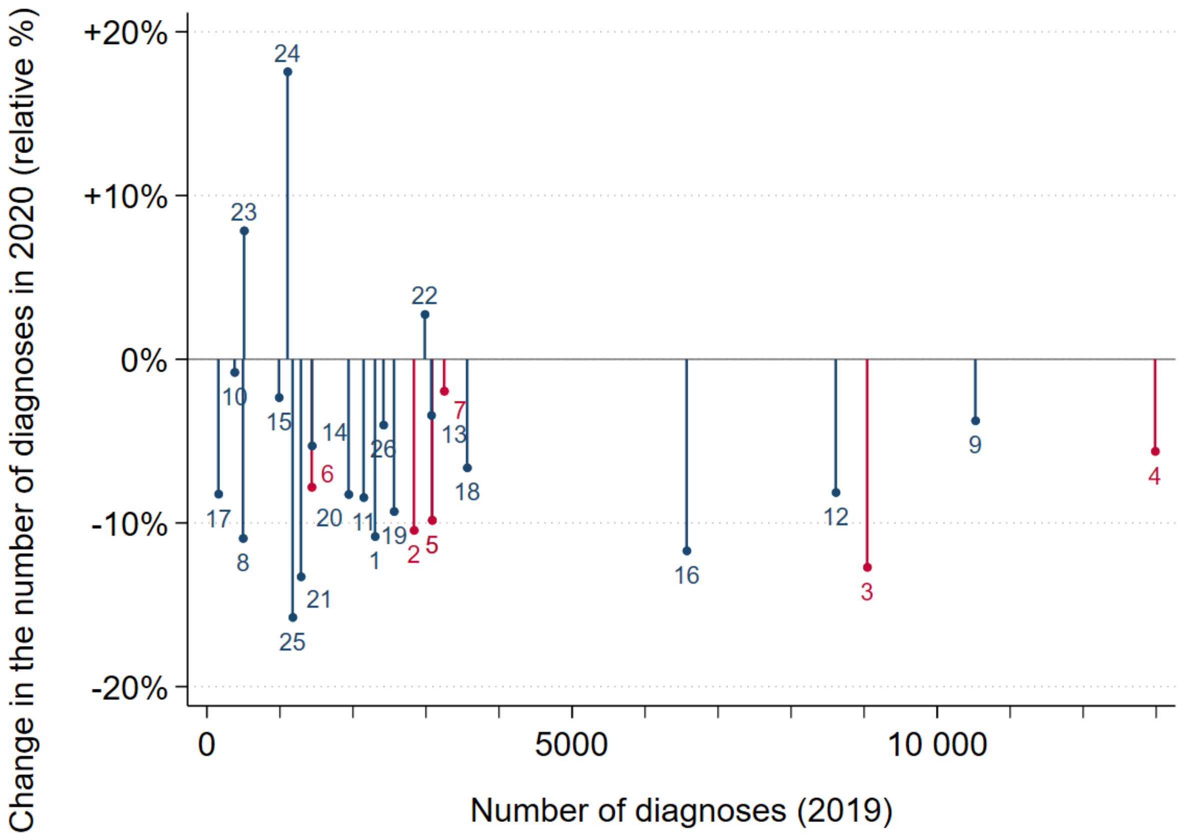

(1)	Oral cavity & pharynx	(16)	Prostate
(2)	Esophagus	(17)	Testis
(3)	Stomach	(18)	Bladder
(4)	Colorectum	(19)	Kidney & other urinary tract
(5)	Liver & intrahepatic bile ducts	(20)	Brain & central nervous system
(6)	Gallbladder & other/unspecified biliary tract	(21)	Thyroid
(7)	Pancreas	(22)	Malignant lymphoma
(8)	Larynx	(23)	Multiple myeloma
(9)	Lung & bronchus	(24)	Leukemia
(10)	Bone & soft tissue	(25)	Other hematologic malignancies
(11)	Skin	(26)	Others
(12)	Breast		
(13)	Cervix uteri		
(14)	Corpus uteri		
(15)	Ovary		

S2 Fig. Number of diagnoses in 2019 and relative change in 2020 (reference: 2019) by cancer site in the CanReCO project, Osaka, Japan.

Red drop lines indicate cancer of the six digestive organs. Blue drop lines indicate cancers in other sites. The total number of diagnoses in the CanReCO project (all sites combined) was 86 857 in 2019 and 80 869 in 2020 (excluding records with second opinions, relative change -6.9%).
