# Supplementary material for: Diagnosis and treatment of digestive cancers during COVID-19 in Japan: A Cancer Registry-based Study on the Impact of COVID-19 on Cancer Care in Osaka (CanReCO)
Source: PLoS One. 2022 Sep 20;17(9):e0274918. doi: 10.1371/journal.pone.0274918 (PMC9488819; doi:10.1371/journal.pone.0274918)
Supplement: S3 Fig — (PDF) [file pone.0274918.s003.pdf]

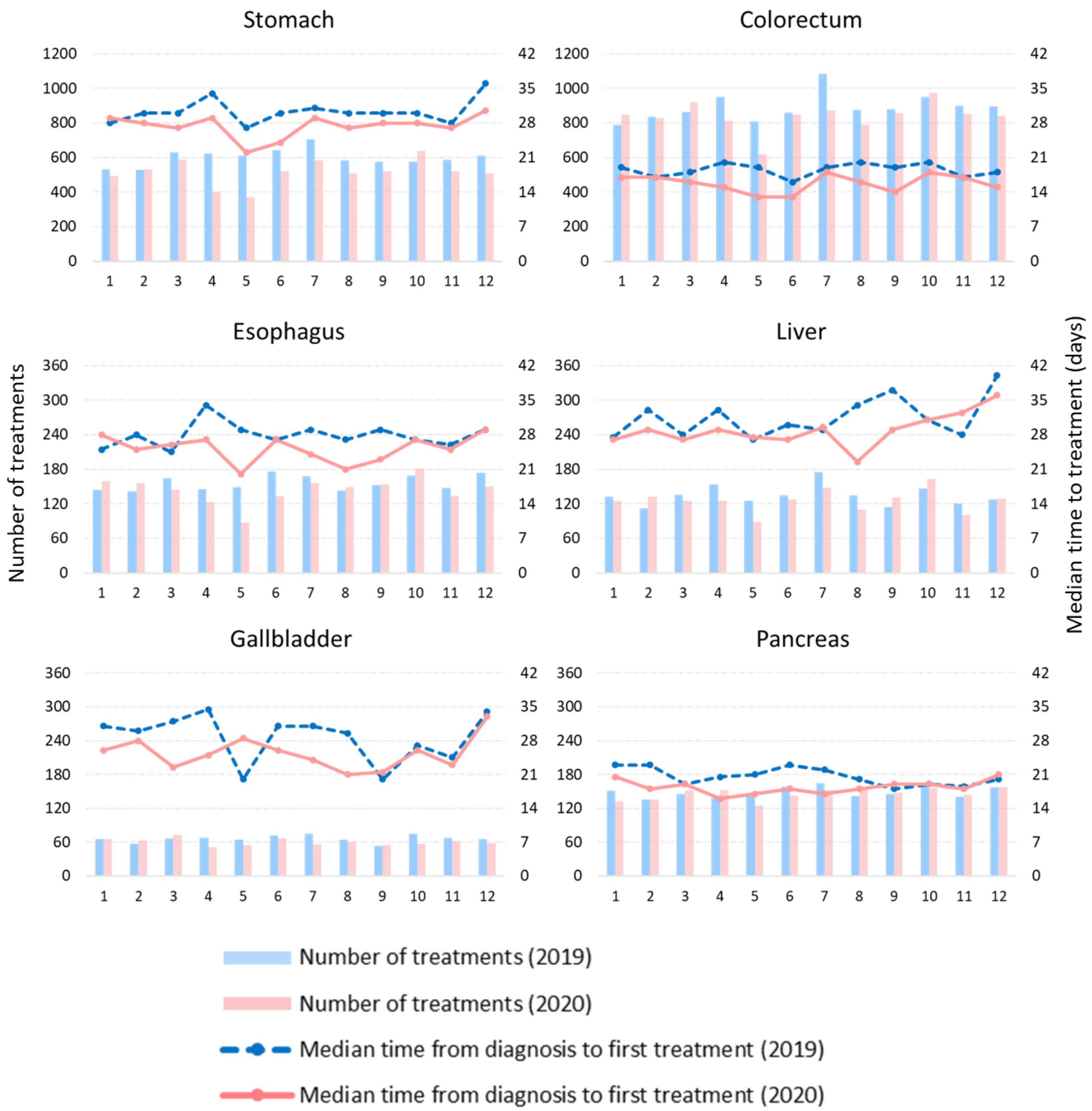

S3 Fig. Number of treatments and median time from diagnosis to first treatment by month of diagnosis for six digestive cancers in the CanReCO project, Osaka, Japan, 2019 and 2020.

First treatment is defined as whichever treatment procedure is carried out first among operation, endoscopic surgery, chemotherapy or radiotherapy. Records with other treatments (e.g., locoregional therapy for liver cancer) and non-invasive treatments (diagnosis/monitoring or palliative care only) were not included in the number of treatments and when deriving time to first treatment, because these treatments have no information on date of treatment initiation. Note that the left axis (number of treatments) is different for stomach and colorectal cancer.
