# Supplementary material for: Diagnosis and treatment of digestive cancers during COVID-19 in Japan: A Cancer Registry-based Study on the Impact of COVID-19 on Cancer Care in Osaka (CanReCO)
Source: PLoS One. 2022 Sep 20;17(9):e0274918. doi: 10.1371/journal.pone.0274918 (PMC9488819; doi:10.1371/journal.pone.0274918)
Supplement: S1 Table — (PDF) [file pone.0274918.s004.pdf]

S1Table. Number of diagnoses and clinical stage 0 & I, percentage of clinical stage 0 & I and relative change by month for six digestive cancers, Osaka, Japan, 2019 and 2020.

	Stomach				Colorectum				Esophagus			
	Number of stage 0 & I / Number of diagnoses (%)		Relative change		Number of stage 0 & I / Number of diagnoses (%)		Relative change		Number of stage 0 & I / Number of diagnoses (%)		Relative change	
Month of diagnosis	Year of diagnosis		Total	Stage 0 & I	Year of diagnosis		Total	Stage 0 & I	Year of diagnosis		Total	Stage 0 & I
	2019	2020			2019	2020			2019	2020		
January	379/691 (54.9)	364/622 (58.5)	-10.0%	-4.0%	351/959 (36.6)	381/1012 (37.7)	+5.5%	+8.5%	95/197 (48.2)	121/239 (50.6)	+21.3%	+27.4%
February	377/677 (55.7)	379/674 (56.2)	-0.4%	+0.5%	390/1024 (38.1)	386/1003 (38.5)	-2.1%	-1.0%	98/197 (49.8)	101/209 (48.3)	+6.1%	+3.1%
March	444/777 (57.1)	394/721 (54.7)	-7.2%	-11.3%	359/1047 (34.3)	402/1098 (36.6)	+4.9%	+12.0%	104/244 (42.6)	97/235 (41.3)	-3.7%	-6.7%
April	441/791 (55.8)	274/531 (51.6)	-32.9%	-37.9%	406/1145 (35.5)	358/993 (36.1)	-13.3%	-11.8%	101/229 (44.1)	91/193 (47.2)	-15.7%	-9.9%
May	414/765 (54.1)	247/508 (48.6)	-33.6%	-40.3%	358/1002 (35.7)	240/795 (30.2)	-20.7%	-33.0%	115/226 (50.9)	55/135 (40.7)	-40.3%	-52.2%
June	447/784 (57.0)	366/708 (51.7)	-9.7%	-18.1%	362/1050 (34.5)	323/1047 (30.9)	-0.3%	-10.8%	109/272 (40.1)	96/203 (47.3)	-25.4%	-11.9%
July	487/863 (56.4)	385/723 (53.3)	-16.2%	-20.9%	476/1308 (36.4)	325/1063 (30.6)	-18.7%	-31.7%	124/264 (47.0)	105/226 (46.5)	-14.4%	-15.3%
August	422/743 (56.8)	328/631 (52.0)	-15.1%	-22.3%	406/1061 (38.3)	295/955 (30.9)	-10.0%	-27.3%	101/226 (44.7)	92/209 (44)	-7.5%	-8.9%
September	382/714 (53.5)	364/668 (54.5)	-6.4%	-4.7%	354/1082 (32.7)	344/1070 (32.2)	-1.1%	-2.8%	121/222 (54.5)	96/221 (43.4)	-0.5%	-20.7%
October	393/729 (53.9)	445/801 (55.6)	+9.9%	+13.2%	393/1132 (34.7)	366/1172 (31.2)	+3.5%	-6.9%	116/270 (43.0)	125/259 (48.3)	-4.1%	+7.8%
November	403/744 (54.2)	394/675 (58.4)	-9.3%	-2.2%	393/1097 (35.8)	340/1029 (33.0)	-6.2%	-13.5%	109/221 (49.3)	97/196 (49.5)	-11.3%	-11.0%
December	441/764 (57.2)	358/632 (56.7)	-17.3%	-18.8%	366/1078 (34.0)	342/1019 (33.6)	-5.5%	-6.6%	122/267 (45.7)	103/214 (48.1)	-19.9%	-15.6%

Clinical stage defined by the UICC (Union for International Cancer Control). The reference for the relative change in 2020 was 2019.

Continued from **S1 Table.**

	Liver				Gallbladder				Pancreas			
	Number of stage 0 & I / Number of diagnoses (%)		Relative change		Number of stage 0 & I / Number of diagnoses (%)		Relative change		Number of stage 0 & I / Number of diagnoses (%)		Relative change	
Month of diagnosis	Year of diagnosis		Total	Stage 0 & I	Year of diagnosis		Total	Stage 0 & I	Year of diagnosis		Total	Stage 0 & I
	2019	2020			2019	2020			2019	2020		
January	90/261 (34.5)	90/224 (40.2)	-14.2%	0.0%	12/110 (10.9)	13/107 (12.2)	-2.7%	+8.3%	67/249 (26.9)	73/254 (28.7)	+2.0%	+9.0%
February	89/221 (40.3)	105/239 (43.9)	8.1%	+18.0%	15/100 (15.0)	18/116 (15.5)	+16.0%	20.0%	64/239 (26.8)	72/248 (29.0)	+3.8%	+12.5%
March	105/268 (39.2)	98/251 (39.0)	-6.3%	-6.7%	20/127 (15.8)	9/140 (6.4)	+10.2%	-55.0%	54/252 (21.4)	63/265 (23.8)	+5.2%	+16.7%
April	118/288 (41.0)	90/227 (39.7)	-21.2%	-23.7%	11/129 (8.5)	10/113 (8.9)	-12.4%	-9.1%	54/282 (19.2)	71/282 (25.2)	0.0%	+31.5%
May	102/268 (38.1)	77/184 (41.9)	-31.3%	-24.5%	17/116 (14.7)	17/93 (18.3)	-19.8%	0.0%	67/266 (25.2)	62/244 (25.4)	-8.3%	-7.5%
June	94/247 (38.1)	111/245 (45.3)	-0.8%	18.1%	18/125 (14.4)	20/120 (16.7)	-4.0%	+11.1%	69/263 (26.2)	61/270 (22.6)	+2.7%	-11.6%
July	118/296 (39.9)	120/273 (44.0)	-7.8%	+1.7%	18/135 (13.3)	15/114 (13.2)	-15.6%	-16.7%	92/308 (29.9)	69/272 (25.4)	-11.7%	-25.0%
August	98/274 (35.8)	86/211 (40.8)	-23.0%	-12.2%	9/123 (7.3)	14/113 (12.4)	-8.1%	+55.6%	67/284 (23.6)	60/268 (22.4)	-5.6%	-10.4%
September	84/241 (34.9)	100/227 (44.1)	-5.8%	+19.0%	15/109 (13.8)	13/94 (13.8)	-13.8%	-13.3%	53/271 (19.6)	69/254 (27.2)	-6.3%	+30.2%
October	102/243 (42.0)	111/276 (40.2)	13.6%	+8.8%	17/118 (14.4)	21/117 (18.0)	-0.8%	+23.5%	84/290 (29.0)	78/288 (27.1)	-0.7%	-7.1%
November	94/233 (40.3)	75/203 (37.0)	-12.9%	-20.2%	11/122 (9.0)	11/99 (11.1)	-18.9%	0.0%	76/271 (28.0)	70/258 (27.1)	-4.8%	-7.9%
December	99/244 (40.6)	85/221 (38.5)	-9.4%	-14.1%	15/121 (12.4)	14/97 (14.4)	-19.8%	-6.7%	59/274 (21.5)	77/283 (27.2)	+3.3%	+30.5%

Clinical stage defined by the UICC (Union for International Cancer Control). The reference for the relative change in 2020 was 2019.
