# Supplementary material for: Diagnosis and treatment of digestive cancers during COVID-19 in Japan: A Cancer Registry-based Study on the Impact of COVID-19 on Cancer Care in Osaka (CanReCO)
Source: PLoS One. 2022 Sep 20;17(9):e0274918. doi: 10.1371/journal.pone.0274918 (PMC9488819; doi:10.1371/journal.pone.0274918)
Supplement: S2 Table — (PDF) [file pone.0274918.s005.pdf]

S2 Table. Median time from diagnosis to first treatment for six digestive cancers in the CanReCO project, Osaka, Japan, 2019 and 2020.

	Median time to treatment in days (IQR)		Relative change*	p-value#
	Year of diagnosis			
Cancer site	2019	2020		
Stomach	30 (18 to 47)	28 (16 to 43)	-6.7%	p<0.001
Colorectum	19 (0 to 33)	15 (0 to 29)	-21.1%	p<0.001
Esophagus	28 (17 to 46)	25 (14 to 42)	-10.7%	p<0.001
Liver	30 (18 to 48)	29 (17 to 43)	-3.3%	0.022
Gallbladder	29 (12 to 46)	25 (12 to 42)	-13.8%	0.059
Pancreas	20 (11 to 35)	18 (10 to 29)	-10.0%	0.001

Abbreviations: IQR, interquartile range. * Relative change of the point estimates in median time to treatment. # p-value of the Wilcoxon rank sum test.
