# Supplementary material for: Diagnosis and treatment of digestive cancers during COVID-19 in Japan: A Cancer Registry-based Study on the Impact of COVID-19 on Cancer Care in Osaka (CanReCO)
Source: PLoS One. 2022 Sep 20;17(9):e0274918. doi: 10.1371/journal.pone.0274918 (PMC9488819; doi:10.1371/journal.pone.0274918)
Supplement: S3 Table — (PDF) [file pone.0274918.s006.pdf]

S3 Table. Number of operations and median time to operation by month for six digestive cancers in the CanReCO project, Osaka, Japan, 2019 and 2020.

	Number of operations			Median time to operation (days)		
	Year of diagnosis		Relative change	Year of diagnosis		Relative change
Calendar month	2019	2020		2019	2020	
Stomach						
January	247	238	-3.6%	34	32	-5.9%
February	242	248	+2.5%	34	34	0.0%
March	280	278	-0.7%	34	31	-10.3%
April	271	185	-31.7%	41	29	-29.3%
May	278	168	-39.6%	29	20	-31.0%
June	296	212	-28.4%	31	28	-9.7%
July	318	263	-17.3%	34	30	-11.8%
August	261	234	-10.3%	36	28	-22.2%
September	261	205	-21.5%	34	27	-20.6%
October	247	284	+15.0%	34	30	-11.8%
November	255	209	-18.0%	35	27	-22.9%
December	259	239	-7.7%	38	33	-13.2%
Colorectum						
January	478	498	+4.2%	26	27	+3.8%
February	502	504	+0.4%	26	25	-3.8%
March	527	532	+0.9%	26	24	-9.6%
April	627	458	-27.0%	28	23	-17.9%
May	495	408	-17.6%	25	19	-24.0%
June	543	539	-0.7%	24	18	-25.0%
July	661	564	-14.7%	27	23	-14.8%
August	551	522	-5.3%	27	21	-22.2%
September	560	513	-8.4%	27	20	-25.9%
October	602	618	+2.7%	26	24	-7.7%
November	560	556	-0.7%	27	25	-7.4%
December	536	497	-7.3%	29	25	-13.8%
Esophagus						
January	39	56	+43.6%	73	67	-8.9%
February	37	48	+29.7%	63	76	+19.8%
March	49	48	-2.0%	74	75	+1.4%
April	38	39	+2.6%	75	66	-12.0%
May	43	28	-34.9%	68	57	-16.2%
June	56	43	-23.2%	76	62	-17.9%
July	50	52	+4.0%	64	73	+13.3%
August	52	44	-15.4%	66	60	-9.1%
September	45	62	+37.8%	74	64	-14.2%
October	50	63	+26.0%	63	73	+16.8%
November	53	50	-5.7%	80	76	-5.6%
December	43	46	+7.0%	80	72	-10.0%

Number of operations excludes records with no information on date of operation. Number of days in time to treatment was rounded up to integer. Relative change for time to treatment was calculated with the crude number of days before rounding up the numbers.

Continued from **S3 Table.**

	Number of operations			Median time to operation (days)		
	Year of diagnosis		Relative change	Year of diagnosis		Relative change
Calendar month	2019	2020		2019	2020	
Liver						
January	57	51	-10.5%	40	39	-2.5%
February	52	62	+19.2%	39	40	+2.6%
March	66	47	-28.8%	36	36	+1.4%
April	71	56	-21.1%	37	34	-9.5%
May	65	50	-23.1%	38	31	-19.7%
June	64	68	+6.3%	44	30	-31.0%
July	83	71	-14.5%	37	39	+5.4%
August	60	53	-11.7%	46	32	-29.7%
September	50	65	+30.0%	43	38	-11.6%
October	73	80	+9.6%	37	36	-4.1%
November	60	53	-11.7%	45	40	-10.1%
December	63	68	+7.9%	44	41	-6.8%
Gallbladder						
January	45	43	-4.4%	31	26	-16.1%
February	38	48	+26.3%	27	33	20.4%
March	49	51	+4.1%	35	27	-22.9%
April	46	41	-10.9%	39	25	-35.1%
May	49	40	-18.4%	27	27	0.0%
June	50	48	-4.0%	31	30	-1.6%
July	51	41	-19.6%	34	27	-20.6%
August	44	34	-22.7%	30	29	-1.7%
September	39	34	-12.8%	28	21	-25.0%
October	51	42	-17.6%	29	27	-6.9%
November	46	41	-10.9%	30	22	-26.7%
December	47	36	-23.4%	35	34	-2.9%
Pancreas						
January	71	72	+1.4%	35	61	+72.9%
February	73	66	-9.6%	35	46	+31.4%
March	53	64	+20.8%	33	42	+27.3%
April	57	73	+28.1%	41	49	+19.5%
May	64	59	-7.8%	36	42	+18.3%
June	75	57	-24.0%	40	68	+70.0%
July	86	61	-29.1%	43	65	+51.2%
August	65	71	+9.2%	50	62	+24.0%
September	68	70	+2.9%	50	51	+2.0%
October	75	64	-14.7%	47	45	-5.3%
November	57	71	+24.6%	54	70	+29.6%
December	62	70	+12.9%	53	63	+18.9%

Number of days in time to treatment was rounded up to integer. Relative change for time to treatment was calculated with the crude number of days before rounding up the numbers.
