# Supplementary material for: Diagnosis and treatment of digestive cancers during COVID-19 in Japan: A Cancer Registry-based Study on the Impact of COVID-19 on Cancer Care in Osaka (CanReCO)
Source: PLoS One. 2022 Sep 20;17(9):e0274918. doi: 10.1371/journal.pone.0274918 (PMC9488819; doi:10.1371/journal.pone.0274918)
Supplement: S4 Table — (PDF) [file pone.0274918.s007.pdf]

S4 Table. Number of endoscopic surgeries and median time to endoscopic surgery by month for three digestive cancers in the CanReCO project, Osaka, Japan, 2019 and 2020.

	Number of endoscopic surgeries			Median time to endoscopic surgery (days)		
	Year of diagnosis		Relative change	Year of diagnosis		Relative change
Calendar month	2019	2020		2019	2020	
Stomach						
January	242	229	-5.4%	31	33	+8.2%
February	252	241	-4.4%	32	32	+1.6%
March	292	260	-11.0%	35	30	-14.3%
April	296	184	-37.8%	41	37	-9.8%
May	274	171	-37.6%	34	28	-17.6%
June	298	251	-15.8%	35	28	-18.8%
July	323	269	-16.7%	35	33	-5.7%
August	275	240	-12.7%	32	30	-6.3%
September	265	267	+0.8%	36	32	-11.1%
October	263	307	+16.7%	34	34	0.0%
November	282	268	-5.0%	30	32	+6.7%
December	302	234	-22.5%	42	37	-13.1%
Colorectum						
January	293	363	+23.9%	0	0	0.0%
February	333	339	+1.8%	0	0	0.0%
March	326	375	+15.0%	0	0	0.0%
April	324	341	+5.2%	0	0	0.0%
May	301	208	-30.9%	0	0	0.0%
June	327	302	-7.6%	0	0	0.0%
July	403	289	-28.3%	0	0	0.0%
August	326	257	-21.2%	0	0	0.0%
September	324	330	+1.9%	0	0	0.0%
October	339	324	-4.4%	0	0	0.0%
November	338	299	-11.5%	0	0	0.0%
December	348	336	-3.4%	0	0	0.0%
Esophagus						
January	73	78	+6.8%	34	42	+22.1%
February	74	84	+13.5%	40	37	-6.3%
March	74	70	-5.4%	32	36	+12.5%
April	70	63	-10.0%	48	37	-22.9%
May	77	40	-48.1%	41	30	-26.8%
June	80	63	-21.3%	39	36	-6.5%
July	84	73	-13.1%	35	30	-13.0%
August	70	65	-7.1%	41	34	-16.0%
September	83	60	-27.7%	36	32	-12.5%
October	80	94	+17.5%	40	39	-2.5%
November	70	62	-11.4%	42	40	-4.8%
December	88	79	-10.2%	41	36	-12.2%

Number of endoscopic surgeries excludes records with no information on date of endoscopic surgery. Number of days in time to treatment was rounded up to integer. Relative change for time to treatment was calculated with the crude number of days before rounding up the numbers.
