# Supplementary material for: Diagnosis and treatment of digestive cancers during COVID-19 in Japan: A Cancer Registry-based Study on the Impact of COVID-19 on Cancer Care in Osaka (CanReCO)
Source: PLoS One. 2022 Sep 20;17(9):e0274918. doi: 10.1371/journal.pone.0274918 (PMC9488819; doi:10.1371/journal.pone.0274918)
Supplement: S5 Table — (PDF) [file pone.0274918.s008.pdf]

S5 Table. Number of screen-detected cases by age group for stomach and colorectal cancer in the CanReCO project, Osaka, Japan, 2019 and 2020.

	Stomach				Colorectum			
	Number of screen-detected / Number of diagnoses* (%)		Relative change		Number of screen-detected / Number of diagnoses (%)		Relative change	
	Year of diagnosis		Total*	Screen-detected	Year of diagnosis		Total*	Screen-detected
Age group	2019	2020			2019	2020		
0–39	15/61 (24.6)	13/48 (27.1)	-21.3%	-13.3%	36/158 (22.8)	24/141 (17.0)	-10.8%	-33.3%
40–49	59/208 (28.4)	43/175 (24.6)	-15.9%	-27.1%	180/561 (32.1)	162/519 (31.2)	-7.5%	-10.0%
50–64	365/1224 (29.8)	239/981 (24.4)	-19.9%	-34.5%	635/2415 (26.3)	631/2326 (27.1)	-3.7%	-0.6%
65–74	509/2978 (17.1)	398/2518 (15.8)	-15.4%	-21.8%	901/4204 (21.4)	676/3700 (18.3)	-12.0%	-25.0%
75–84	224/3268 (6.9)	179/2895 (6.2)	-11.4%	-20.1%	482/3765 (12.8)	426/3691 (11.5)	-2.0%	-11.6%
85–	34/921 (3.7)	34/938 (3.6)	+1.8%	0.0%	65/1140 (5.7)	97/1211 (8.0)	+6.2%	+49.2%

The reference for the relative change in 2020 was 2019. * The total number of diagnoses excludes records with unknown routes to diagnosis. The age grouping in S5 Table followed the cut-off age for screening (≥50 years old for stomach cancer and ≥ 40 years old for colorectal cancer). Thus, the figures for the relative change in total are different from those (relative change by age group) in Table 1.
