# Supplementary material for: Diagnosis and treatment of digestive cancers during COVID-19 in Japan: A Cancer Registry-based Study on the Impact of COVID-19 on Cancer Care in Osaka (CanReCO)
Source: PLoS One. 2022 Sep 20;17(9):e0274918. doi: 10.1371/journal.pone.0274918 (PMC9488819; doi:10.1371/journal.pone.0274918)
Supplement: Attachment — Submitted filename: Response to Reviewers.docx [file pone.0274918.s009.docx]

**Response to Reviewers**

**Journal Requirements**

When submitting your revision, we need you to address these additional requirements.

1. Please ensure that your manuscript meets PLOS ONE's style requirements, including those for file naming. The PLOS ONE style templates can be found at 
https://journals.plos.org/plosone/s/file?id=wjVg/PLOSOne_formatting_sample_main_body.pdf and 
https://journals.plos.org/plosone/s/file?id=ba62/PLOSOne_formatting_sample_title_authors_affiliations.pdf

Thank you for your guidance. We have amended the manuscript to meet PLOS ONE’s style requirements.

2. Please provide additional details regarding participant consent. In the ethics statement in the Methods and online submission information, please ensure that you have specified what type you obtained (for instance, written or verbal, and if verbal, how it was documented and witnessed). If your study included minors, state whether you obtained consent from parents or guardians. If the need for consent was waived by the ethics committee, please include this information.

Once you have amended this/these statement(s) in the Methods section of the manuscript, please add the same text to the “Ethics Statement” field of the submission form (via “Edit Submission”).

Thank you for providing the guidance. We have included additional details in the main text as follows (Line 82-85), and added the same text to the “Ethics Statement” field of the submission form.

“Patient consent was waived by the Board because data for the CanReCO project has been collected for health policy planning and research use. For research use of the data, the Board approved an opt-out approach using a written consent form for each DCCH.”

For additional information about PLOS ONE ethical requirements for human subjects research, please refer to http://journals.plos.org/plosone/s/submission-guidelines#loc-human-subjects-research.

3. In your Data Availability statement, you have not specified where the minimal data set underlying the results described in your manuscript can be found. PLOS defines a study's minimal data set as the underlying data used to reach the conclusions drawn in the manuscript and any additional data required to replicate the reported study findings in their entirety. All PLOS journals require that the minimal data set be made fully available. For more information about our data policy, please see http://journals.plos.org/plosone/s/data-availability.

Upon re-submitting your revised manuscript, please upload your study’s minimal underlying data set as either Supporting Information files or to a stable, public repository and include the relevant URLs, DOIs, or accession numbers within your revised cover letter. For a list of acceptable repositories, please see http://journals.plos.org/plosone/s/data-availability#loc-recommended-repositories. Any potentially identifying patient information must be fully anonymized.

Important: If there are ethical or legal restrictions to sharing your data publicly, please explain these restrictions in detail. Please see our guidelines for more information on what we consider unacceptable restrictions to publicly sharing data: http://journals.plos.org/plosone/s/data-availability#loc-unacceptable-data-access-restrictions. Note that it is not acceptable for the authors to be the sole named individuals responsible for ensuring data access.

We will update your Data Availability statement to reflect the information you provide in your cover letter.

Thank you for the guidance. We have updated our Data Availability Statement as follows.

“All relevant data are within the manuscript and its Supporting Information files.”

4. We note that you have included the phrase “data not shown” in your manuscript. Unfortunately, this does not meet our data sharing requirements. PLOS does not permit references to inaccessible data. We require that authors provide all relevant data within the paper, Supporting Information files, or in an acceptable, public repository. Please add a citation to support this phrase or upload the data that corresponds with these findings to a stable repository (such as Figshare or Dryad) and provide and URLs, DOIs, or accession numbers that may be used to access these data. Or, if the data are not a core part of the research being presented in your study, we ask that you remove the phrase that refers to these data.

We have added an additional Supporting Information file to describe the manuscript (S5 Table), and all relevant data are now provided within the paper. As stated in the previous section, Data Availability Statement was changed accordingly.

5. Please include a separate caption for each figure in your manuscript.

A section on “Separate caption for each figure” was added after the References and before the section on Supporting Information.

6. Please review your reference list to ensure that it is complete and correct. If you have cited papers that have been retracted, please include the rationale for doing so in the manuscript text, or remove these references and replace them with relevant current references. Any changes to the reference list should be mentioned in the rebuttal letter that accompanies your revised manuscript. If you need to cite a retracted article, indicate the article’s retracted status in the References list and also include a citation and full reference for the retraction notice.

Thank you for your guidance. We have reviewed our reference list. We confirm that it is complete and correct, and we do not include papers that have been retracted. Please note that the following citations were added to the revised manuscript.

39. World Health Organization. Therapeutics and COVID-19: Living guideline, 14 July 2022. Geneva: 2022 (WHO/2019-nCoV/therapeutics/2022.4). Licence: CC BY-NC-SA 3.0 IGO.

40. Altmann DM, Boyton RJ. COVID-19 vaccination: The road ahead. Science. 2022;375(6585):1127-32. doi: 10.1126/science.abn1755.

43.	Okuyama A, Watabe M, Makoshi R, Takahashi H, Tsukada Y, Higashi T. Impact of the COVID-19 pandemic on the diagnosis of cancer in Japan: analysis of hospital-based cancer registries. Jpn J Clin Oncol. 2022. doi: 10.1093/jjco/hyac129.

**Response to reviewers**

**5. Review Comments to the Author**

Please use the space provided to explain your answers to the questions above. You may also include additional comments for the author, including concerns about dual publication, research ethics, or publication ethics. (Please upload your review as an attachment if it exceeds 20,000 characters)

**Reviewer #1**: Dear Editor, thank you so much for inviting me to revise this manuscript.

This study addresses a current topic.

The manuscript is quite well written and organized. English could be improved.
Figures and tables are comprehensive and clear.
The introduction explains in a clear and coherent manner the background of this study.

Thank you for taking the time to review our manuscript. We appreciate your constructive feedback on the manuscript.

We suggest the following modifications:
• Introduction section: although the authors correctly included important papers in this setting, we believe a couple of studies should be cited within the introduction ( PMID: 35109688; PMID: 32658591 ), only for a matter of consistency. We think it might be useful to introduce the topic of this interesting study.

Thank you for your comment. We have added a phrase on the topic and cited relevant articles in the Discussion section (Line 319-320) as follows.

“Proper allocation of resources should be planned to prepare for the foreseen backlog to avoid further delay. The situation of the pandemic has been continuously changing with the evolution of variant strains and the development of treatment and vaccines [39, 40]. The COVID-19 pandemic continues across Japan, and the CanReCO project will continue to monitor cancer care and its outcome. The full impact of COVID-19 on cancer will be revealed only after a few years.”

• Methods and Statistical Analysis: nothing to add.

Thank you for your comment.

• Discussion section: Very interesting and timely discussion. Of note, the authors should expand the Discussion section, including a more personal perspective to reflect on. For example, they could answer the following questions – in order to facilitate the understanding of this complex topic to readers: what potential does this study hold? What are the knowledge gaps and how do researchers tackle them? How do you see this area unfolding in the next 5 years? We think it would be extremely interesting for the readers.

Thank you for pointing it out. We have added a phrase to mention the potential of our study, knowledge gaps and futures in this area in the Discussion section (Line 291-296).

“Although the real consequences of the COVID-19 pandemic are yet to be known, we consider that measuring its impact on cancer care uncovers the stability, strengths and weaknesses of the healthcare system in a country. In addition to our results, understanding patients’ experiences during the pandemic and examining both short- and long-term cancer survival may provide insights into which point of care can be improved to achieve better cancer outcomes.”

However, we think the authors should be acknowledged for their work. In fact, they correctly addressed an important topic, the methods sound good and their discussion is well balanced.

One additional little flaw: the authors could better explain the limitations of their work, in the last part of the Discussion.

Thank you for your comment. We have added some phrases to better explain the limitations of our study in the Discussion section (Line 359-363, 369-371).

“Our results on the relative change in the number of operations were comparable to the estimates derived from the National Clinical Database encompassing all surgical procedures in Japan [45]; therefore, we assume our results are valid. Nonetheless, our results may not be generalizable to other countries or other areas in Japan. It is because the scale and timing of the pandemic, healthcare system, and the baseline characteristics of a population, including socioeconomic situations, health-seeking behaviours and screening uptakes, may differ originally between and within countries.”

“The final limitation is that information on elective or emergency operation, socioeconomic status, comorbidities and measures of performance status has been not linked to these results. Identifying the affected populations and assessing the detailed situation is a key to proper reallocation of resources and aligning supply with patients’ needs. More detailed data on these characteristics that can be derived from DPC data are now available and will be able to reveal which populations were most affected by COVID-19.”

We believe this article is suitable for publication in the journal although major revisions are needed. The main strengths of this paper are that it addresses an interesting and very timely question and provides a clear answer, with some limitations.
We suggest a linguistic revision and the addition of some references for a matter of consistency. Moreover, the authors should better clarify some points.

Thank you for your comment. A professional British language editor proofread the manuscript. We have modified some points for clarification.

**Reviewer #2:** The authors analyzed GI cancer diagnosis and treatment patterns in Osaka, Japan, comparing the COVID pandemic and the time before. Since the impact of the COVID pandemic is different according to the medical environment of each country, the data in this paper will help compare the situation of each country.

We would like to thank you for taking the time to review our manuscript. Thank you for your encouraging comment.
